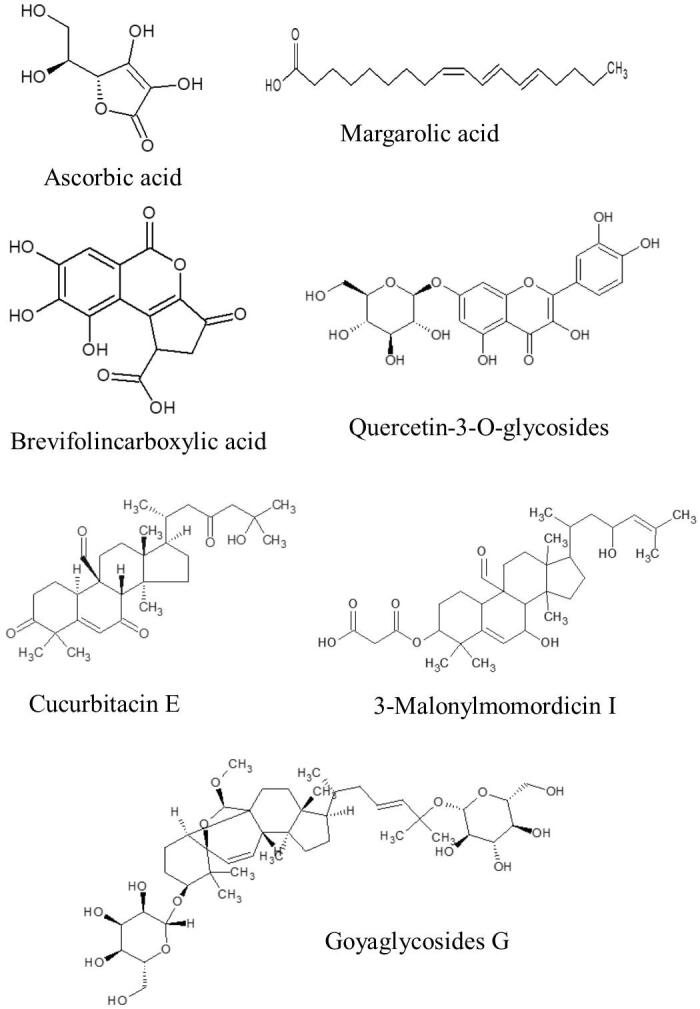# Antioxidants profile of *Momordica charantia* fruit extract analyzed using LC-MS-QTOF-based metabolomics

**DOI:** 10.1016/j.fochms.2021.100012

**Published:** 2021-01-26

**Authors:** Vikneswari Perumal, Alfi Khatib, Qamar Uddin Ahmed, Bisha Fathamah Uzir, Faridah Abas, Suganya Murugesu, Mohd Zuwairi Saiman, Riesta Primaharinastiti, Hesham El-Seedi

**Affiliations:** a Department of Pharmaceutical Technology, Faculty of Pharmacy & Health Sciences, Universiti Kuala Lumpur Royal College of Medicine Perak, 30450 Ipoh, Perak Darul Ridzuan, Malaysia; b Department of Pharmaceutical Chemistry, Kulliyyah of Pharmacy, International Islamic University Malaysia, Kuantan 25200, Pahang Darul Makmur, Malaysia; c Laboratory of Natural Products, Institute of Bioscience, Universiti Putra Malaysia, Serdang 43400, Malaysia; d Institute of Tropical Agriculture and Food Security (ITAFoS), University Putra Malaysia, 43400 Serdang, Selangor Darul Ehsan, Malaysia; e Institute of Biological Sciences, Faculty of Science, University of Malaya, Kuala Lumpur 50603, Malaysia; f Faculty of Pharmacy, Airlangga University, Surabaya 60155, Indonesia; g Division of Pharmacognosy, Department of Medicinal Chemistry, Uppsala University, Biomedical Centre, Box 574, SE-75123 Uppsala, Sweden

**Keywords:** Antioxidants, DPPH, FRAP, LC-MS-QTOF, Metabolomics, *Momordica charantia*

## Abstract

*Momordica charantia* fruit is claimed to have healthy benefit. Despite this potential claim, the phytochemical study of this fruit is still lacking. Thus, this study aimed to evaluate the antioxidants profile of *Momordica charantia* (Cucurbitaceae) fruit. The antioxidant activity of the ethanolic extracts of various polarities was evaluated and the metabolites that are responsible for its activity were identified using metabolomics approach. Six different mixture of ethanol in water that are 0%, 20%, 40%, 60%, 80%, and 100% (v/v) was extracted using dveseeded fruit sample. Liquid chromatography-mass spectrometry-quadrupole time of flight and multivariate data analysis was used to identify the metabolites that were either antioxidants or pro-oxidants. The 80% ethanol extract exhibited the most antioxidant activity when tested in both 2, 2-diphenyl-1-picrylhydrazyl (DPPH) and ferric reducing antioxidant power (FRAP) antioxidant assays. This extract showed the most intense LC-MS signals represented to ascorbic acid, margarolic acid, brevifolincarboxylic acid, quercetin 3-O-glycoside, kuguacin H, cucurbitacin E, 3-malonylmomordicin I, and goyaglycoside G correlating to the anti-oxidant activity. This study reports for the first time the existence of brevifolincarboxylic acid in this fruit, and the antioxidant activity of 3-malonylmomordicin I and goyaglycoside G. In addition, the loading plots revealed the unknown compounds possessing the antioxidant activity which are potential to be isolated in the future study.

## Highlights

• The 80% ethanol extract of *M. charantia fruit* exhibited the most antioxidant activity.
Brevifolincarboxylic acid is a new antioxidant compound reported in *Momordica charantia* fruit.
3-Malonylmomordicin I and goyaglycoside G are possessing anti-oxidant activity.

## Graphical abstract

**Figure:**
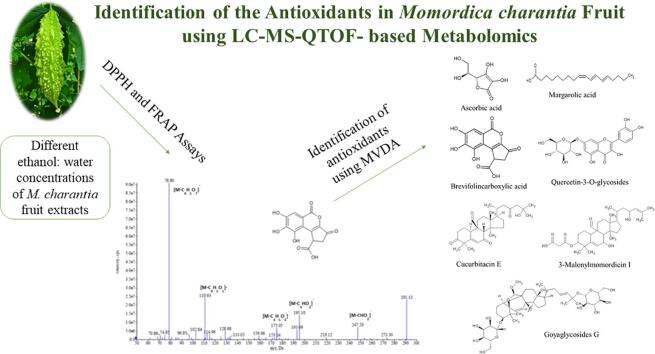

## 1 Introduction

Antioxidants play a crucial role in the pathogenesis of disorders that leads to severe health effects such as diabetes mellitus (DM), coronary heart disease, neurodegenerative disorders, cancers, Alzheimer’s disease, and hepatotoxicity. An elevated level of reactive oxygen species (ROS) in the human body is a natural consequence of aerobic metabolism and is essential for maintaining the oxygen homeostasis in tissues. However, ROS can potentially damage essential macromolecules resulting in carcinogenic effects or lead to cardiovascular diseases. The human body requires adequate amounts of antioxidants to fight against ROS such as the hydroxyl radical (OH.), the superoxide anion (O^2–^) and non-radical molecules such as hydrogen peroxide (H_2_O_2_), nitric oxide (NO), and singlet oxygen, among others (Karthivashan, Tangestani, Arulselvan, Abas, & Fakurazi, 2013).

Currently, most people have suboptimal diets due to their busy lifestyles and try to compensate by consuming dietary supplements with antioxidants such as propyl gallate, butylated hydroxytoluene and butylated hydroxyanisole. These synthetic antioxidant supplements, however, have been to the subject of increasing regulatory scrutiny because of their toxicity when used over extended periods. Because of this, natural food and food-derived antioxidants are more attractive as chemo-preventive agents against oxidative damage (Karthivashan et al., 2013).

Medicinal plants are often rich in natural antioxidants and contain various other bioactive compounds that could have promising health effects. *Momordica charantia* is a popular medicinal plant of the Cucurbitaceae family that is widely available in local markets. It is known as ‘peria katak’ in Malaysia and bitter melon or bitter gourd in English. Wu and Ng reported that it is an antioxidant-rich vegetable; it has been reported to possess antidiabetic, antimicrobial, antiviral and anticancer activities (Grover, Yadav, & S. P., 2004).

Several compounds including charantin, momorcharin, charine, cryptoxanthin, diosgenin, gentisic acid, momorcharasides, momordenol, momordicilin, momordicin, momordicinin, momordin, momordicosides, polypepide-p, rosmarinic acid, taraxerol, treshalose, vicine and zeaxanthin have been isolated from this plant. The hypoglycemic properties of the plant have been attributed to momorcharin, polypeptide-p and vicine which generally exert insulin-like effects. Some of the metabolites possessing antioxidant effects isolated and identified from the fruit includes rosmarinic acid, trehalose, vicine momordicine, phenols (catechin, epicatechin and chlorogenic, gallic and gentisic acids), flavonoids (rutin and naringin) and ferulic acid (Kumar, Kavimani, & Jayaveera, 2011). However, no investigations related to the correlation between the mixture of antioxidants in this plant and its bio- activity. All previous studies reported based on the antioxidant activities of the individual compounds. The bioactivity of a compound may differ when it presents within a complex matrix such as plant material. For example, the antioxidant activity of a sample can easily disappear when it is fractionated, and this phenomenon is attributed to possible synergistic effects with other components in the sample (Kuhlisch & Pohnert, 2015). Thus, the reported antioxidant compounds may not be solely responsible for the antioxidant activity of the whole fruit. One way of determining all the constituents related to any bioactivity observed is by applying the holistic approach called metabolomics. Metabolomics approach relatively detects all metabolites present in a sample matrix and their correlation with the tested bioactivity using multivariate data analysis (Verpoorte et al., 2009). Roos, Roseler, Buter, and Simmen (2004), who correlated the NMR profiles of St John’s wort with its pharmacological activity have reported an excellent example of this approach.

The analytical platform used in this study was liquid chromatography-mass spectrometry-quadrupole time of flight (LC-MS-QTOF), a technique increasingly being used for metabolomics studies. It provides the high resolution needed to separate the components of complex biological mixtures as well as high sensitivity, at a relatively low cost (Trammell & Charles, 2013). Therefore, the hypothesis of this study is LCMS-based metabolomics approach able to identify antioxidants responsible for the FRAP and DPPH activities from *M. charantia* using an unsupervised multivariate data analysis.

Besides that, study relating to solvent polarity and composition are lacking on the antioxidant extraction. In order to determine the optimal solvent polarity to extract all potential compounds corresponding to the biological activity observed, different polarity and/or composition of solvent will be used. Thus, this study aimed to evaluate the antioxidants profile of *Momordica charantia* (Cucurbitaceae) fruit extracted using solvent containing ethanol and water with different polarity. Generally, polar solvents are preferred in the recovering of phytoconstituents majorly phenols. Ethanol is reportedly sought as a good solvent for bioactive components from plant matrices. The most suitable solvents are aqueous mixtures with other solvents including ethanol, methanol, acetone and ethyl acetate (Do et al., 2014). Technically, this study used different composition of ethanol and water for extraction and thus evaluate the effects of solvent polarity on the antioxidant activity of *M. charantia* fruit extracts followed by identification of the metabolites related to this activity extracted from different composition of ethanol and water incorparted with LC-MS-QTOF-based metabolomics.

## 2 Materials and methods

### 2.1 Chemicals

Analytical reagents; acetic acid, acetone, acetone and hydrochloric acid were purchased from R & M Marketing (Essex, UK). The standard reagents and solutions; sodium acetate, pyridine, ascorbic acid, 2,2-diphenyl-1-picrylhydrazyl (DPPH) of 95% potency, 2,4,6-tris (2-pyridyl)-s-triazine (TPTZ) of 99% potency, iron (III) chloride hexahydrate; N-methyl-N- (trimethylsilyl) trifluoroacetamide (MSTFA) of 97% potency and methoxyamine hydrochloride of 98% potency brand were Sigma-Aldrich (St. Louis, MO., USA).

### 2.2 Plant material collection and extraction

The fruits of *M. charantia* were harvested from a local farm located in Perak, Malaysia. The identification voucher number PIIUM 0215A was provided by the Herbarium Kulliyyah of Pharmacy, International Islamic University of Malaysia. As for extraction, twelve-week old fruit samples collected randomly from the farm. The seeds removed, and the fruit flesh was washed and lyophilized in liquid nitrogen. After freeze-drying, the fruit flesh was ground to a fine powder and kept at −80 °C until further processing.

The extraction of fruit flesh carried out using an ethanol–water solvent mixture composed of six different polarities following an established method (Javadi et al., 2014). Five milligrams of the powdered freeze-dried fruit flesh was transferred into a conical flask together with 150 mL of each ethanol–water mixture (0%, 20%, 40%, 60%, 80% or 100%), and followed by sonication for 30 min. Filtration using filter papers (No. 1 Whatman International, Maidstone, UK) was done after immediately after sonification Later the extracts were recovered using a rotary evaporator (Buchi, Flawil, Switzerland) and the temperature was maintained at 40 ± 1 °C. The freeze-dried extracts were stored at −80 °C until further analysis.

### 2.3 DPPH radical scavenging activity

The assay is one of the routine antioxidant assays. The *M. charantia* fruit extracts analyzed for the radical scavenging activity following Karthivashan et al. (2013) with slight modifications. The aliquot of the extract prepared by dissolving two milligrams of the extract in one millilitre of distilled water. Accurately, 20 µL of the sample aliquot and ascorbic acid (control) was added to 80 µL of DPPH solution and incubated in the dark at room temperature for 10 min, followed by the measurement of absorbance using an instrument named, microplate reader (Tecan, Männedorf, Switzerland) at the wavelength of 540 nm. A blank solution prepared by mixing 20 µL of solvent (water) with 80 µL of DPPH solution. The DPPH radical scavenging activity (percentage) of the samples calculated using the following equation:

(1) $$Percentage\left(\%\right)=[\left(OD_{control}-OD_{sample}\right)/\left(OD_{control}\right]x100\%$$

The calibration curve was plotted based radical scavenging activity (%) vs various dilutions of sample/standard assay. The radical scavenging (%) was obtained from the half-maximal inhibition concentration (IC_50_) values of DPPH radical scavenging activity. The analysis was experimented by using six replication. Final values were calculated based on the average and the standard error of the mean (SEM).

### 2.4 FRAP (Ferric reducing antioxidant power) assay

The reducing power of the *M. charantia* fruit extracts was determined using the ferric reducing antioxidant assay following the method adapted from Karthivashan et al. (2013). Firstly, the FRAP reagent was prepared fresh by mixing 2.5 mL of TPTZ solution (10 mM) with 2.5 mL of FeCl_3_ solution (20 mM) and 25 mL of acetate buffer (0.1 M, pH 3.6). The reagent mixture kept under incubation at the temperature of 37 °C for 10 min at. Approximately, 20 μL of extract aliquots and ascorbic acid (positive control), transferred into 96-well plate and added with 140 μL of distilled water. The sample mixture was then treated with the FRAP reagent by adding 40 μL of it into each sample well that produces blue-coloured solutions. Meanwhile, a blank solution was containing 40 μL of FRAP reagents in 200 μL of distilled water were added to the microplate as well. The mixtures were then incubated in the dark at room temperature for 20 min and later using a microplate reader (Tecan, Männedorf, Switzerland) the absorbance was measured at the wavelength of 593 nm. A calibration curve has been generated based on the absorbances obtained from serial dilutions of the standard. Lastly, the ferric reducing power was determined by interpolation of the absorbance measured using the standard calibration curve, and the value was expressed as ascorbic acid equivalents per gram sample (AAE μg/g).

### 2.5 LC-MS-QTOF testing

A LC-MS-QTOF system was chosen to analyze samples following Lawal et al. (2016) with some modifications. By dissolving about 2 mg of each extract in 80% v/v ethanol, the sample aliquots were preapred. Accurately, 200 µL of the sample aliquot was filtered using microfilter and transferred into a glass vial. One microliter of the sample aliquot was injected on an Agilent RRHD Eclipse Plus C18 column of 2.1 × 50 mm length and 1.8 µm internal c Q-TOF mass spectrometry using the parameters listed below.

## 3 Parameters

Flow rate: 0.3 mL/min (10 min)
Eluant: Absolute methanol
Q-TOF MS: Electrospray ionization (ESI)
Ionization Mode: negative and positive
Capillary voltages: 3 Kv
Sampling cone voltages: 30 V
Desolvation flow: 700 L/h at the temperature 300 °C
Source temperature: 110 °C

The raw data were obtained from mass-to-charge ratio (*m*/*z*) range between 100 and 1000 at 0.2 and 0.02 s scan time and an interscan delay, respectively. The 556.2771 Da in ESI positive mode, 200 mol of leucine-enkephalin was used as the lock spray (flow rate: 3 µL/min, 10 s frequency) to ensure accuracy and reproducibility. MS/MS spectra were obtained by a collision energy ramp of 10 to 30 eV.

ACD/Spec Manager v.12.00 (Advanced Chemistry Development, Inc., ACD/Labs Toronto, Canada) was used for the analysis of LCMS data. The raw (*.xms) files were converted to netCDF (*.cdf) files using the same software. The files were proceeded to preprocessing, peak extraction, alignment adjustment and retention time correction with XCMS. The XCMS package in R version 2.15.1 (www.bioconductor.org) computed using basic commands applying XCMS’s default settings (http://masspec.scripps.edu/xcms/documentation.php). Peaks listed were then exported to Microsoft Excel (Microsoft, Redmond, WA) in the form of *.txtfile and peaks were identified based on the average area, corrected retention time and *m*/*z* ratio data.

### 3.1 Statistical analysis

The preprocessed LC-MS-QTOF and antioxidant activity data were transferred into the SIMCA P + 14.0 software (Umetrics AB, Umeå, Sweden) for chemometric analysis (MVDA). In order to obtain the best discrimination of the sample according to the antioxidant activity and the LC-MS-QTOF profile, Orthogonal partial least square (OPLS) model was used. The results were UV scaled and centred to allow OPLS modelling. One-way ANOVA with a Tukey comparison at a confidence interval of 95% using Minitab 14 (Minitab Inc., State College, PA., USA) was done to determine the significant differences.

## 4 Results and discussion

### 4.1 DPPH radical scavenging activity

The stable DPPH free radical has an unpaired valence electron on one atom of its nitrogen bridge, and one of the most popular assays of antioxidant activity that measures the scavenging capacity for this radical. Scavenging capacity will determine the potency of the antioxidant compounds to reducing the delocalization of unpaired electron on the DPPH radical by donating a hydrogen atom, thus turns the vibrant violet colour of the reagent to a colourless solution (Sharma & Tej, 2009). The radical scavenging activity of DPPH displayed a concentration-dependent result. Table 1 shows the DPPH radical scavenging capacities of the extracts. The values are expressed as IC_50_ values (concentration of fruit ethanolic extract inhibiting radical generation 50%). The extracts obtained with 80% ethanol exhibited the strongest inhibition: 0.37 mg/mL (p < 0.05) contrasted to 0.02 mg/mL of ascorbic acid. The extract obtained with 20% ethanol showed the lowest activity. The present finding is comparable to Suhailah et al. (2011). They observed that an aqueous extract of *M. charantia* fruit exhibited the weakest DPPH free radical scavenging activity. The difference in IC_50_ values among the *M. charantia* extracts has been attributed to differences in the metabolic profiles obtained with solvents of different polarities.

**Table 1:** Comparison of the yield of extractions, FRAP value and DPPH radical scavenging activities of *Momordica charantia* fruit.

Extract (% of ethanol in water, v/v) and positive control	FRAP (AAE μg of ascorbic acid/g) (mean ±SEM)	DPPH IC_50_ (mg/mL) (mean ± SEM)	Yield of Extraction (%)
0	54.27±0.60^d^	1.10±0.08^a^	40.36 ± 3.23^b^
20	65.32±0.23^c^	1.10±0.04^a^	38.51 ± 1.10^b^
40	85.51±1.04^b^	1.09±0.08^a^	23.30 ± 1.26^d^
60	86.11±2.16^b^	1.05±0.10^ab^	35.32 ± 2.30^bc^
80	113.85±0.94^a^	0.37±0.07^d^	62.03 ± 1.27^a^
100	112.31±1.34^a^	0.53±0.06^cd^	24.71 ± 3.14^cd^
Ascorbic acid	114.58±1.73^a^	0.02±0.01^e^	ND

Values represent the mean ± standard error of the mean (SEM), n = 6.

Value in each column with different subscript letter are significantly (p < 0.05 different using ANOVA followed by Tukey’s test.

ND: Not determined.

### 4.2 Ferric reducing activity in the FRAP assay

The FRAP assay is another routine quantitative test with high-throughput that measures the antioxidant capacity via reduction action. The mechanism involves the reduction of ferric iron (Fe3+) to ferrous ion (Fe2+) by antioxidant compounds present in the extract, which observed through the formation of an intense blue colour (Rajurkar & Hande, 2011). The antioxidant-rich extracts exhibit their reducing power by donating a hydrogen atom that substantially breaks the free radical chain. Results generated using the standard calibration are tabulated (Table 1) below. The 80% ethanolic extract had the highest antioxidant capacity (113.85 AAE μg/g). This value is comparably close to that of standard (114.58 AAE μg/g). The value of 80% ethanolic extract were followed sequentially by the extracts obtained with absolute ethanol, 60% ethanol, 40% ethanol, 20% and water with 112.31, 86.11, 85.51, 65.32 and 54.27 AAE μg/g, respectively. Notably, the ferric reducing capabilities of the *M. charantia* extracts contribute more to their antioxidant activity than their free radical scavenging properties.

The 80% ethanol extract was the most active in both the DPPH and FRAP assays with the highest extraction yield (62.03%) (see Table 1), while the less active extract (40% ethanol) had the lowest (23.26%). This yield is an advantage for commercialization since the active extract is the most abundant.

### 4.3 Multivariate data analysis

**Fig. 1:**
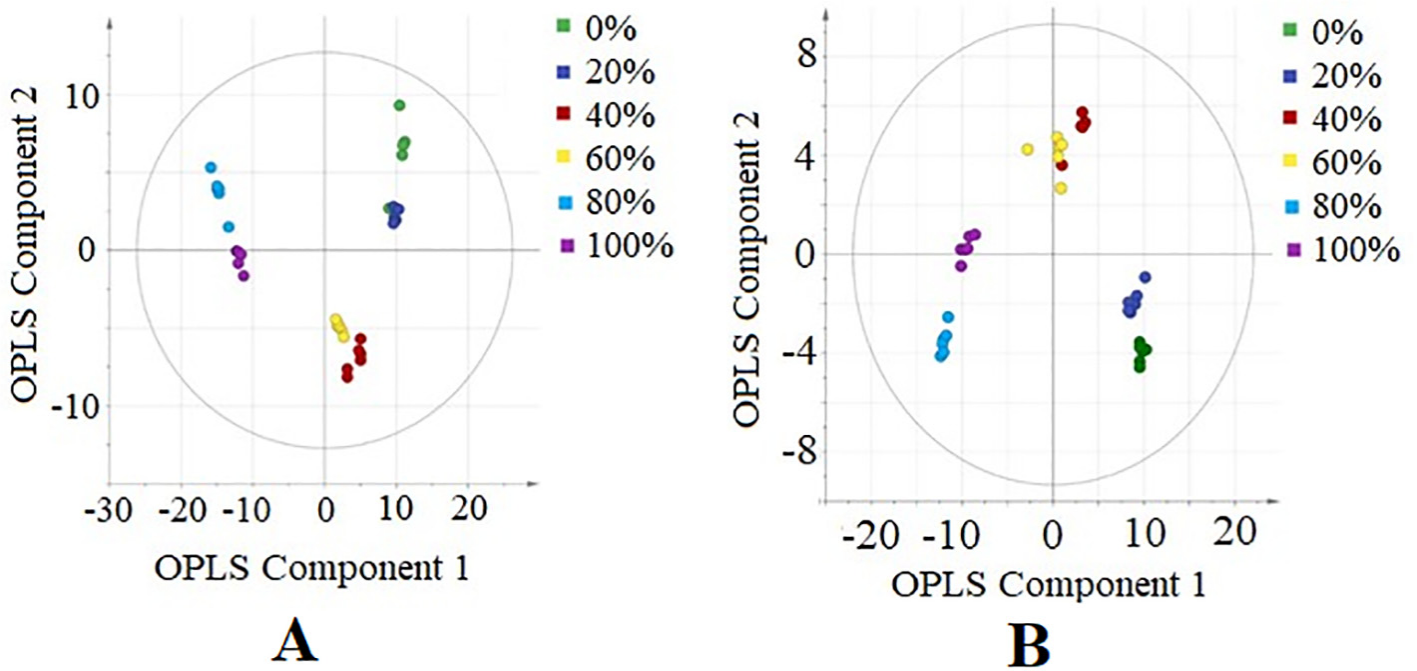
Score scatter plot for the OPLS model of different extracts of *M. charantia* fruit (0, 20, 40, 60, 80, and 100% v/v ethanolic extracts) analyzed using LC-MS-QTOF with in negative (A) and positive (B) ion modes.

The chemical profiles of different ethanolic extracts of *M. charantia* fruit were correlated to their antioxidant activities (FRAP and DPPH radical scavenging activities) using OPLS analysis with the UV scaling. This method was chosen in this study since it gives each variable a variance numerically equal to its standard deviation (Eriksson, Trygg, & Wold, 2008). The samples were discriminated based on the X data matrix (the metabolite profile) and the Y data matrix (the antioxidant activities). Score scatter plot of the extracts was illustrated in Fig. 1(A) and (B) using LC-MS in negative and positive ion modes, respectively. Both plots showed apparent separation that discriminates the active and inactive *M. charantia* extracts based on component 1 of the OPLS model, where the active extracts found to be clustered in the negative quadrant. The maximum variation explained by OPLS component 1 and 2 from LC-MS negative ion mode were 39.6 and 9.3%, respectively. The maximum variation explained by OPLS component 1 and 2 from LC-MS in positive ion mode were 42.6 and 7.6%, respectively.

**Fig. 2:**
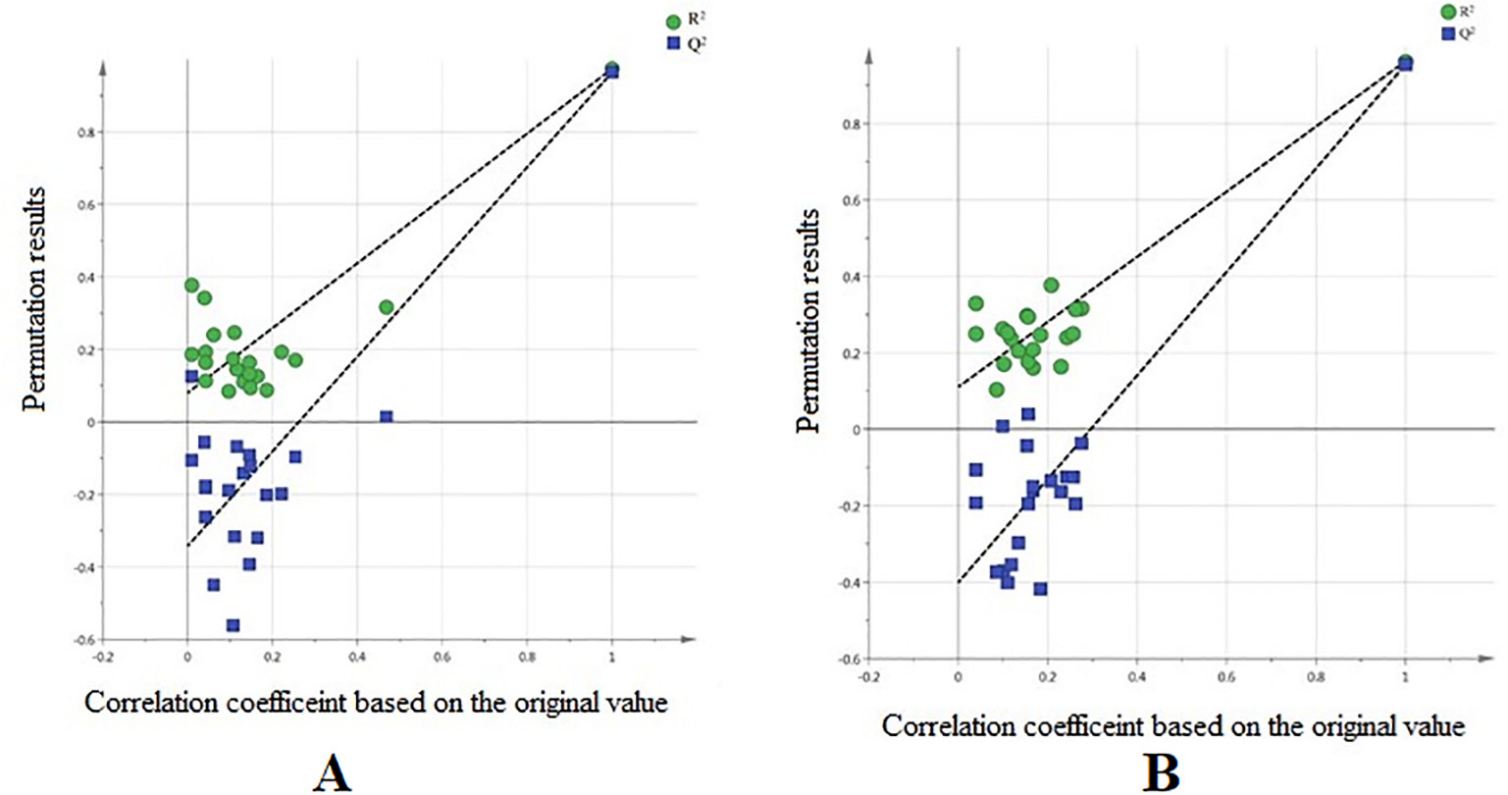
Permutation test for OPLS model of LC-MS-QTOF in negative ion mode (A). The intercepts of R^2^Y and Q^2^Y were 0.08 and −0.34, respectively. (B) Permutation test for the OPLS model of LC-MS-QTOF in positive ion mode. The intercepts of R^2^Y and Q^2^Y were 0.11 and −0.40, respectively.

Both OPLS models were validated using a permutation test. In this analysis, the goodness of fit (R^2^Y) and predictive ability (Q^2^Y) were determined for cross-validation to select the most suitable model. The intercepts of R^2^Y and Q^2^Y for the LC-MS data collected negative ion mode were 0.08 and −0.34, respectively (Fig. 2(A)). The intercepts of R^2^Y and Q^2^Y for the LC-MS data obtained from the positive ion mode were 0.11 and −0.40, respectively (Fig. 2(B)). The results are validated through the intercepts values of R^2^Y and Q^2^Y which should be below 0.3 and −0.05, respectively (Eriksson et al., 2008).

**Fig. 3:**
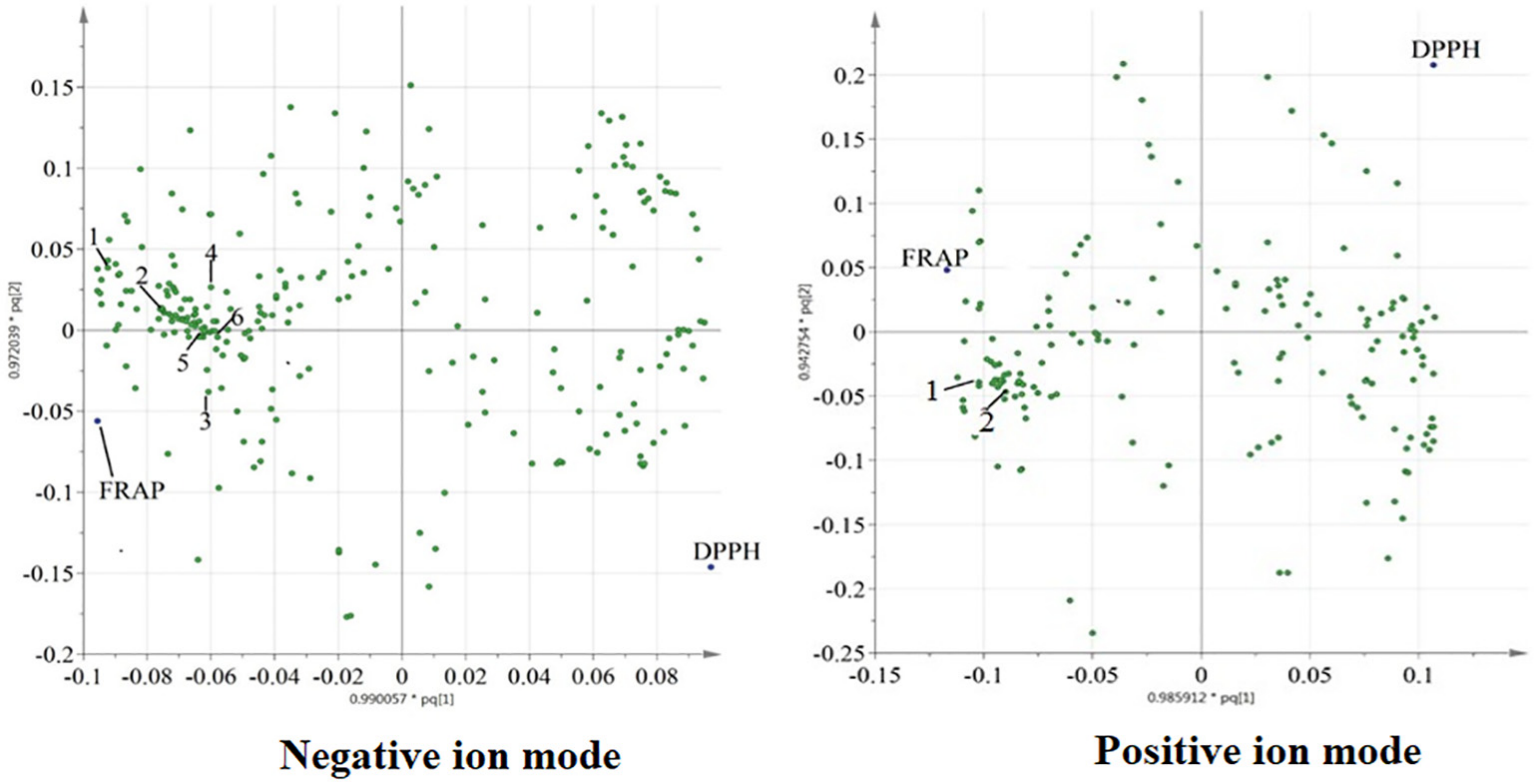
The loading scatter plot of the OPLS model of the extracts identified using LC-MS-QTOF in (A) negative [(1) ascorbic acid, (2) margarolic acid, (3) brevifolincarboxylic acid, (4) quercetin 3-*O*-glycoside, (5) kuguacin H and (6) cucurbitacin E] and (B) positive ion mode [(1) 3-malonylmomordicin I, and (2) goyaglycoside G].

The antioxidants identified using LC-MS in negative ion mode were (1) ascorbic acid, (2) margarolic acid, (3) brevifolincarboxylic acid, (4) quercetin 3-O-glycoside, (5) kuguacin H, and (6) cucurbitacin E (Fig. 3A). The antioxidants identified using LC-MS in positive ion mode were (1) 3-malonylmomordicin I, and (2) goyaglycoside G (Fig. 3B). Other spots in Fig. 3B which were close to the FRAP spot and far to the DPPH spot represented the unknown antioxidant compounds that are potential to be isolated in the future study.

**Supplementary figure 1:**
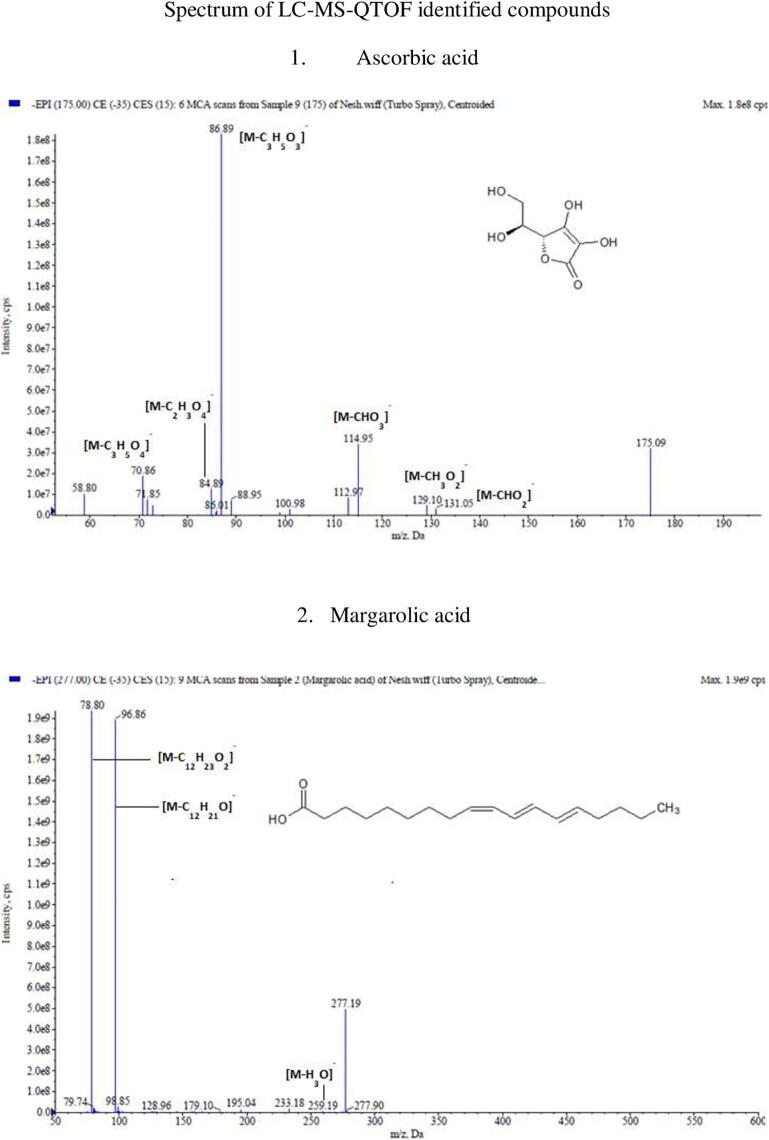


Table 2, Table 3 show the antioxidants tentatively identified based on the fragmentation of the parent ions of each compound using LC-MS/MS in negative and positive ion modes, respectively. The recognition of each antioxidants was confirmed by referring the databases/published articles with experimental data of compounds having antioxidant activity, including ACD Lab, Inc. (Toronto, Canada). The chemical structures of these antioxidants are displayed in Supplementary Fig. 1.

**Table 2:** Antioxidants tentatively identified in the aqueous ethanolic extracts of *M. charantia* fruit based on MS^2^ fragmentation in negative ion mode.

No	[M−H]^-^	MS^2^ fragments ions	Tentative metabolites	References
1	174.92	[M−C_3_H_5_O_4_]^-^ at *m*/*z* 71, [M−C_2_H_3_O_4_]^-^ at *m*/*z* 85, [M−C_3_H_5_O_3_]^-^ at *m*/*z* 87, [M−CHO_3_]^-^ at *m*/*z* 115, [M−CH_3_O_2_]^-^ at /z 129, and [M−CHO_2_]^-^ at *m*/*z* 131	Ascorbic acid	(Frenich, Torres, Vega, Vidal, & Bolaños, 2005)
2	277.22	[M−C_12_H_23_O_2_]^-^ at *m*/*z* 79, [M−C_12_H_21_O]^-^ at *m*/*z* 97, and [M−H_3_O]^-^ at *m*/*z* 259	Margarolic acid	(FoodDB 012466)
3	291.13	[M−C_8_H_5_O_7_]^-^ at *m*/*z* 79, [M−C_8_H_5_O_5_]- at *m*/*z* 111, [M−C_4_H_5_O_4_]^-^ at *m*/*z* 175, [M−C_4_HO_3_]^-^ at *m*/*z* 195, and [M−CHO_2_]^-^ at *m*/*z* 247	Brevifolincarboxylic acid	(Kumar, Singh, & Kumar, 2017)
4	463.09	[M−C_12_H_15_O_8_]^-^ at *m*/*z* 177, [M−C_12_H_13_O_7_]^-^ at *m*/*z* 195, [M−C_11_H_13_O_6_]^-^ at *m*/*z* 223, and [M−C_6_H_13_O_6_]^-^ at *m*/*z* 283	Quercetin-3-O-glycoside	(Keskes et al., 2017)
5	483.11	[M−C_23_H_31_O_3_]^-^ at *m*/*z* 129, [M−C_19_H_29_O_2_]^-^ at *m*/*z* 195, and [M−C_10_H_11_O_3_]^-^ at *m*/*z* 305.	Kuguacin H	(Chen et al., 2009)
6	555.30	[M−C_21_H_29_O_5_]^-^ at *m*/*z* 195, and [M−C_10_H_11_O_3_]^-^ at *m*/*z* 377	Cucurbitacin E	(FoodDB 014623)

**Table 3:** Antioxidants tentatively identified in the aqueous ethanolic extracts of *M. charantia* fruit based on MS^2^ fragmentation in positive ion mode**.**

No	[M + H]^+^	MS^2^ fragments ions	Tentative metabolites	References
1	559.19	[M−C_30_H_46_O_3_] ^+^ at *m*/*z* 104 [M−C_7_H_13_O_5_]^+^ at *m*/*z* 381, [M−C_4_H_10_]^+^ at *m*/*z* 500, and [M−HO]^+^ at *m*/*z* 541,	3-Malonylmomordicin I	(Mekuria, Takerio, Shin-ichi, & Chul-Sa, 2005)
2	811.59	[M−C_31_H_53_O_13_] ^+^ at *m*/*z* 177, [M−C_29_H_47_O_10_] ^+^ at *m*/*z* 255, [M−C_26_H_49_O_11_]^+^ at *m*/*z* 273, [M−C_23_H_41_O_7_] ^+^ at *m*/*z* 381, [M−C_8_H_19_O_5_]^+^ at *m*/*z* 615, and [M−HO]^+^ at *m*/*z* 793.	Goyaglycoside G	(FoodDB 017687)

Oxidation reaction is a major process involved in the cellular mechanisms of human system that can be of supportive and destructive at the same time. The reaction leads to the production of pro-oxidants or reactive oxygen species (ROS) as by-products that may lead to oxidative stress or destruction at cellular level. Pro-oxidants produced in continuous metabolic routes are able to cause cellular damages in various mechanism that subsequently leads to numerous oxidative stress related diseases depending on the intensity of the components and its reduction (Neha, Haider, Pathak, & Yar, 2019).

The role of antioxidants are evident in preventing oxidative stress related diseases via counteraction of pro-oxidants. Antioxidants are obtained through daily diets where it is absorbed from the food consumed or from supplements. Synthetic antioxidants are majorly used in processed foods. In past decades, plant antioxidants have drawn much interest among the researchers due to their potential and beneficial effects on human health. The use of synthetic antioxidants in long term may lead to some damaging effect to human enzyme system and DNA (Neha et al., 2019). Therefore, natural antioxidants are considerably reliable in combating ROS and oxidative stress in human system.

*M. charantia* is known for its various pharmacological effects which may have been contributed by its antioxidant compounds (Gülçin, 2012). This study has screened the antioxidant activity of the fruits extract as well as identified the potential metabolites responsible in exerting the antioxidant effects of the fruit. The outcome of the study will help the research personnel to have better understanding on the potential metabolites that causes the pharmacology activity observed. Of all the solvent composition with different polarity range, the 80% ethanolic extract has shown good radical scavenging and reducing action which was produced by the antioxidant compounds in the sample matrix. The polarity of the extract has high influence on the projected bioactivity with alcohol to be one of the best solvent for extraction of wide range of compounds of semi polar and polar nature and to exhibit high antioxidant values (Sun, Wu, Wang, & Zhang, 2015). The 80% ethanol composition in aqueous exhibited great potential to extract compounds with semi-polar to polar side chains especially hydroxyl which is the promising functional group that may responsible for the activity observed (Do et al., 2014). This corresponds to the compounds present in the extract that belongs to triterpene, triterpenoid glycoside, flavonoids, organic and phenolic acids that possess antioxidant properties.

The metabolomics approach based on LC-MS analysis found to be an effective method to profile metabolites, technically in identifying the antioxidants in the *M. charantia* fruit extracts. The ease of the metabolomics approach is that the application of multivariate data analysis will help to identify the potential metabolite(s) causing the bioactivity observed without the need to isolate the individual compound(s) in the first place. In this study, the antioxidants identified from LC-MS based metabolomics were ascorbic acid, margarolic acid, brevifolincarboxylic acid, quercetin-3-O-glycoside, kuguacin H, cucurbitacin E, 3-malonylmomordicin I and goyaglycoside G. This finding is consistent with the previous studies on the antioxidant activities of the single compound(s) individually as stated above. Antioxidant capacity of most plant metabolites are generally proportional to the presence of hydroxyl (OH) group in the ring of the structure (White et al., 2014).

Anilakumar, Phani, and Nallamuthu (2015) reported the presence of ascorbic acid in *M. charantia* fruit. Ascorbic acid or vitamin C is a powerful antioxidant that can be found abundantly in many foods and plants majorly in oranges and lemons (Lawson et al., 2017). Ascorbic acid acts as a radical scavenger by reacting with oxidants by terminating radical chain reactions via single electron transfer (Gülçin, 2012).

Margarolic acid is also known as α-eleostearic acid (conjugated linolenic acid) were previously reported in the *n*-hexane extract of the seeds of *M. charantia* (Potawale et al., 2008). This acid has been studied for its antioxidant activity and has demonstrated to have an *in vitro* activity (Dhar et al., 2007). Suzuki, Abe, and Miyashita (2014) reported that the protective ability of conjugated linolenic acid is mainly due to the fast oxygen absorption to form dimers and polymers. Additionally, its antioxidant activity is due to its high content of trans double bonds (*cis*-9, *trans*-11, *trans*-13-octadecatrienoic) (Saha & Mahua, 2013). Špika, Žanetić, Pinatel, Vitanović, and Strikić (2013) later reported on the presence of margarolic acid (0.09%) in the Levantinka virgin olive oils, however with low abundancy.

Brevifolincarboxylic acid is a phenolic acid with antioxidant activity found in pomegranate leaves (Zhang, Yujiao, Yuanhu, Jing, & Junwei, 2010). The current study reveals for the first time the presence of this compound in *M. charantia* fruit. The antixodant activity of this compound relates to its chemical structure. The aromaticity and high conjugation with multiple hydroxyl groups make phenolic acids good electron or hydrogen atom donors, allowing them to neutralize free radicals and other ROS (Zhang & Tsao, 2016). Besides that, the heterocyclic structure of brevifolincarboxylic acid which is composed of conjugated dienes and polar OH groups suggests that it is able to act as a redox-active agent, with the ability to interact with other reactive species contributing to its antioxidant capacity (White et al., 2014).

Quercetin 3-O-glycoside, also known as isoquercitrin is a flavonoid, reported being present in *M. charantia* (Svobodova et al., 2016). The antioxidant effect of isoquercitrin is due to its phenolic hydroxyl groups at the 3′ - and 4′ positions of the flavonoid B ring and these groups are the key to its radical scavenging activity, while the hydroxyl group at the 7-position of the flavonoid ring A does not involve in the reaction (Shibano, Masahiko, Masahide, & Kimiye, 2008). Yang, Kotani, Arai, and Kusu (2001) further reported that the catechol group in its B ring improves its electron-donating ability and thus facilitates metal chelation and electron delocalization. The compounds can also be found in other medicinal plants including *Bauhinia longifolia*, *Hyptis fasciculate, Etlingera elatior* and many more (dos Santos et al., 2014).

Kuguacin H is a cucurbitane triterpenoid isolated from *M. charantia* fruit by Chen et al. (2009). However, no antioxidant activity were reported for this compound. Kaushik, Aeri, and Mir (2015) reported the presence of cucurbitacin E in *M. charantia*, a distinct compound found in the Cucurbitaceae family, known for the bitter taste. Cucurbitacins are consist of tetracyclic cucurbitane nucleus skeleton pertaining a variety of oxygenation functionalities at different positions with diverse chemical categories. The compound was reported to give desired activity in combination with some commercial drugs especially as anticancer agent (Chanda, Biswas, Kar, & Mukherjee, 2020). Another study by Zha et al. (2015) have reported that the compound is capable of inducing autophagy in human cancer cells via upregulation of AMPK activity. Moreover, Tannin-Spitz, Grossman, Dovrat, Gottlieb, and Bergman (2007) demonstrated that cucurbitacin E exhibits antioxidant properties, probably through the direct scavenging effect of free radicals.

The 3-malonylmomordicin I is another bitter compound found in bitter gourd. Connolly and Robert (2010) reported the presence of 3-malonylmomordicin I in the leaves of *M. charantia*. The compound was reported to be involved in defence against polyphagous insects (Kashiwagi et al., 2007). Lastly, the presence of goyaglycosides G in *M. charantia* fruit was reported by Murakami, Emoto, Matsuda, and Yoshikawa (2001). However, no potential bioactivity were reported previously using these compounds. This study reports for the first time the antioxidant activity of both compounds.

Chemically, the presence of certain functional groups highly influence the antixodant property of the particular compound. The presence of OH group in plant constituents are sought to provide various pharmacological effects based on its position in the structure (White et al., 2014, Chen et al., 2020). Similarly, the presence of OH attached to the heterocyclic structure or the ring of the compound observed, are known to be electron donors that will exert both oxidizing and reducing capacity that potential enhance the reaction with multiple reactive species that corresponds to oxidation reactions. The presence of OH as side chains in a chemical structure can potentially cause antioxidant reaction. Technically, this can be observed in most of the phenolic and flavonoid compounds. Based on this study, brevifolincarboxylic acid and quercetin 3-O-glycoside may have demonstrated their antioxidant activity due to the presence of OH attached to their heterocyclic ring. Hydroxyl group are also found to be apart of the cucurbitacins, 3-malonylmomordicin I and goyaglycosides G structures that may have the similar effects on their functions as antioxidant agents.

Apart from that, carboxylic groups (–COOH) are known to display electron-donating ability due to its conjugation and induction effects. Thus, an electron-donating functional group can increase the electron cloud density in the ring structure that contributes to the reduced dissocation energy in the phenolic hydroxyl bond that subsequently enhance its free radical scavenging activity (Chen et al., 2020). Brevifolincarboxylic acid as discussed above is a phenolic acid possessing a carboxylic group. Overall, the presence of hydroxyl group in the compounds observed may have contributed to their antioxidant activity.

Although, the fruit is being consumed for various reasons, it perhaps have exhibited potential pharmacological activities due to these compounds. Therefore, this study can serve as an additional informative research paper in regards of the potential polarity of the solvent (80% ethanol) to extract optimum antioxidants as discussed that can be further explored as potential antioxidants and repurposed for other health beneficial effects. Apart from that, the isolation and quantification of these unique compounds may enhance the use of the fruit as nutraceutical as well as pharmaceutical components. The study also suggesting that the consumption of *M. charantia* fruit as a dietary functional food will provide an appreciable antioxidant effects.

## 5 Conclusions

The present study revealed the chemical constituents of *M. charantia* that are responsible for the antioxidant activity of using LC-MS metabolomics approach. The 80% ethanol–water combination solvent extract exhibited marked antioxidant activities according to both the DPPH and FRAP assays. The major antioxidant compounds identified include ascorbic acid, margarolic acid, brevifolincarboxylic acid, quercetin 3-O-glycoside, kuguacin H, cucurbitacin E, 3-malonylmomordicin I, and goyaglycoside G. This study reports for the first time the presence of brevifolincarboxylic acid in *M. charantia* fruit, as well as the antioxidant activity of 3-malonylmomordicin I and goyaglycoside G. In addition, this study revealed the suspected unknown antioxidant compounds which are prospective to be purified in the future study.

## Funding

This study was funded by the IIUM for Publication Research Initiative Grant [PRIGS18-027-0027]; RACE funding [14-011-0017] and VR (Stockholm, Sweden) [2016-05908].

## Author contributions

V. Perumal conducted the tests, interpreted the results, wrote the original manuscript, A. Khatib contributed to conceptualization, project administration, validation, review and editing the manuscript, and provided research grant. S. Murugesu collected the experimental data, reviewed and edited the manuscript. Q.U. Ahmed helped in addressing the problems encountered during testing and validated the of the research outcome, reviewing and editing the manuscript. B.F. Uzir and F. Abas reviewed the manuscript, provided research grant, and advised on the methodology of this research. M. Z. Saiman contributed to the resource, and edited and improved the manuscript, R. Primaharinastiti and H. El-Seedi provided funding for the research work, reviewed and edited the manuscript.

## Declaration of Competing Interest

The authors declare that they have no known competing financial interests or personal relationships that could have appeared to influence the work reported in this paper.

## Figures and Tables

**Supplementary figure 2:**